# Exercise positively impacts global longitudinal strain in women at risk of developing cardiovascular disease

**DOI:** 10.1002/ejsc.12047

**Published:** 2024-03-18

**Authors:** James Murray, Hunter Bennett, Eva Bezak, Rebecca Perry

**Affiliations:** ^1^ Allied Health and Human Performance University of South Australia Adelaide South Australia Australia; ^2^ Alliance for Research in Exercise, Nutrition and Activity University of South Australia Adelaide South Australia Australia; ^3^ Cancer Research Institute University of South Australia Adelaide South Australia Australia; ^4^ Department of Physics University of Adelaide Adelaide South Australia Australia

**Keywords:** diabetes, echocardiography, GLS, hypertension, physical activity

## Abstract

Cardiovascular (CV) disease accounts for one third of deaths in females, with hypertension (HTN) and type 2 diabetes mellitus (T2DM) risk factors for its development. Global longitudinal strain (GLS) is a strong prognostic indicator for future CV dysfunction and can be impaired in women with HTN and T2DM. This study aimed to determine if exercise impacts GLS in women with HTN or T2DM. A randomized crossover trial was conducted with 15 women (aged 64.1 ± 4.7) diagnosed with HTN (*n* = 12) or T2DM (*n* = 3). Participants completed an 8‐week exercise and 8‐week non‐exercising control period, separated by a 21.6 ± 14.2‐week washout period. Resting echocardiography and exercise testing were performed pre and post each arm to measure GLS and aerobic fitness (VO_2_peak). GLS (%) improved significantly following exercise (−16.8 ± 1.5 to −18.4 ± 1.8; *p* *≤* 0.001, *d* = 0.98), but not control (−17.2 ± 2.0 to −16.9 ± 1.4; *p* = 0.585, *d* = −0.14). Similarly, VO_2_peak (mL/kg/min) increased following exercise (18.0 ± 2.1 to 19.2 ± 2.6; *p* *≤* 0.001, *d* = 0.53), but not control (17.5 ± 2.7–17.2 ± 2.7; *p* = 0.269, *d* = −0.12). There were significant between‐group differences for GLS (*p* *≤* 0.001, *d* = 1.02) and VO_2_peak (*p* = 0.011, *d* = 0.63). Aerobic exercise positively impacts GLS and VO_2_peak in women with HTN and T2DM. GLS may inform exercise professionals regarding the early effectiveness of an aerobic exercise intervention and infer a reduction in CV disease risk.

## Highlights

- The impact of aerobic exercise on global longitudinal strain (GLS), and other measures of cardiovascular (CV) function, was explored in women with hypertension and type 2 diabetes.
- Eight weeks of aerobic exercise significantly improved GLS (very large effect; *d* = 1.02) and aerobic capacity (moderate effect; *d* = 0.63) compared to control.
- Improvements observed in GLS and aerobic capacity were clinically meaningful. Significant reductions in systolic and diastolic blood pressure were also observed following exercise, but not control.
- GLS can measure the effectiveness of aerobic exercise on myocardial function in women at risk of developing CV disease.

## 1 INTRODUCTION

Cardiovascular (CV) disease is the leading cause of death worldwide (World Health Organisation, 2022). Hypertension (HTN) and type 2 diabetes mellitus (T2DM) are two primary risk factors for the development of CV disease, with their prevalence related to modifiable lifestyle factors, including diet, smoking and physical inactivity (Einarson et al., 2018; Mills et al., 2020). Although incidence is more common among men, women diagnosed with HTN or T2DM are at greater risk of developing CV disease (coronary artery disease, myocardial infarction and stroke) and dysfunction (heart failure), with CV disease accounting for one in three deaths in women globally each year (Einarson et al., 2018; Peters et al., 2014; Ramirez et al., 2018; Vogel et al., 2021; Yusuf et al., 2004). In women with HTN or T2DM, regular monitoring of CV health and targeted interventions are key to prevent the development of CV disease and dysfunction (Williams et al., 2018).

With respect to regular monitoring, echocardiography is recommended as part of CV disease risk assessment in individuals with HTN or T2DM, as the presence of myocardial dysfunction is known to increase the risk of CV mortality (Williams et al., 2018). However, parameters assessed via echocardiography to detect signs of hypertensive or diabetic heart disease, such as left ventricular (LV) diastolic dysfunction, LV mass, LV hypertrophy and LV ejection fraction, only identify abnormalities once they are clinically overt and, in some cases, symptomatic (Ernande et al., 2011). Global longitudinal strain (GLS) is a highly sensitive CV imaging measure that detects early signs of myocardial dysfunction prior to clinical abnormalities arising, and is a strong prognostic indicator for future CV dysfunction (D’Elia et al., 2020; Fortuni et al., 2021). Individuals with HTN or T2DM have been shown to have reduced GLS, despite normal measures of LV ejection fraction and LV diastolic function (Soufi Taleb Bendiab et al., 2017; Tadic et al., 2021), suggesting that it is more sensitive to early impairments in LV function than traditional measures (Ernande et al., 2011; Tadic et al., 2021). Therefore, interventions that positively impact GLS could significantly reduce the risk and prevent the development of future CV disease and dysfunction in women with HTN or T2DM.

Exercise is a well‐recognised intervention for the prevention of CV disease, with strong evidence demonstrating its inverse relationship with CV and all‐cause mortality (Kr et al., 2019; Visseren et al., 2021). Exercise is associated with a reduction in blood pressure and glycated hemoglobin, and is considered first line treatment for the management of HTN and T2DM (Sharman et al., 2019; Williams et al., 2020). Aerobic exercise is also associated with improvements in measures of CV function and fitness, such as stroke volume (SV) and peak oxygen consumption (VO_2_peak) (American College of Sports Medicine, 2013). However, there is limited research investigating the effect of exercise on GLS. In patients with breast cancer undergoing cardiotoxic anti‐cancer therapies, reductions in LV ejection fraction and GLS are common (Lyon et al., 2022; Thavendiranathan et al., 2014). Research investigating the use of exercise to prevent these reductions in LV ejection fraction and GLS during therapy is emerging, but to date, its effect is inconclusive (Murray et al., 2021; Foulkes et al., 2022). A recent systematic review and meta‐analysis performed by the current authors explored the effect of exercise on GLS across a range of healthy and CV compromised populations (Murray et al., 2022). Meta‐analyses of randomized controlled trials demonstrated that exercise positively impacted GLS in individuals post myocardial infarction, but no effect was observed in populations with CV disease risk factors (including HTN and T2DM) (Murray et al., 2022). However, in exercising studies only (no control group), exercise did positively impact GLS in populations at risk of developing CV disease. It is important to note that GLS was not a primary outcome measure in all studies included in this review, which may be explained by its underuse in the echocardiography field, and women were significantly underrepresented when compared to men (only 28% of participants exposed to exercise were female) (Murray et al., 2022). The association between traditional measures used to assess the effectiveness of an exercise intervention (i.e., VO_2_peak) and GLS has also not been explored (Murray et al., 2022). Given the sensitivity of GLS, it may offer a viable measure to determine the acute effectiveness of an exercise intervention on myocardial function, prior to observing changes in VO_2_peak. This information could inform exercise professionals regarding the effectiveness of an exercise intervention in its early stages, ultimately allowing for adjustments to improve future outcomes.

Therefore, the aim of this study was to determine if exercise impacts GLS, and other parameters of myocardial function, in women with HTN or T2DM. Additionally, the association between the changes in GLS and changes VO_2_peak were explored. It was hypothesized that exercise will positively impact GLS in women with HTN or T2DM.

## 2 MATERIALS AND METHODS

### 2.1 Participants and experimental design

This study was a prospective, randomized crossover trial. Participants were eligible for inclusion if they were: female, diagnosed with HTN or T2DM, had no history of structural heart disease or arrythmias and had no physical disability that prevented safe exercise testing and training. Participants were recruited between August 2021 and June 2022, with data collection finalized in December 2022. This study was granted ethics approval by the Central Adelaide Local Health Network Human Research Ethics Committee (protocol number 13976) and the University of South Australia Human Research Ethics Committee (protocol number 203909), and prospectively registered with the Australian New Zealand Clinical Trials Registry (ACTRN12621001030864). All experimental procedures were explained to the participants, with signed, informed consent obtained prior to partaking in any research activities. All procedures conformed to the standards set by the Declaration of Helsinki. Upon enrollment into the study, participants were randomized into one of the two arms: exercise or control, using a simple method of randomization (coin flip). Outcome measures were collected at four timepoints (T) throughout the study. T1 and T2 consisted of baseline and post‐intervention measurements for the initial exercise and control arms, respectively. Both arms had a minimum 4‐week washout period, after which participants crossed over into the opposite arm. T3 and T4 consisted of the same baseline and post‐intervention measurements, respectively, for the crossed over participants.

### 2.2 Measurements

#### 2.2.1 Participant history

The medical history, including years diagnosed with HTN or T2DM, current medications and smoking history were recorded for all participants. Self‐reported physical activity was collected using the International Physical Activity Questionnaire–Short Form (Craig et al., 2003).

#### 2.2.2 Body composition and point of care testing

Participants' height (m) and mass (kg) were measured using a wall stadiometer and calibrated SECA 703 electronic scales (Hamburg, Germany). Body mass index (BMI) was calculated (kg/m^2^). Resting blood pressure was measured using an OMROM HEM 7320 blood pressure monitor (Kyoto, Japan). Non‐fasted blood glucose was measured using an Accu‐Check Performa Metre (Indianapolis, USA). Resting blood pressure and non‐fasted blood glucose of all participants were measured at each timepoint, regardless of their diagnosed comorbidity.

#### 2.2.3 Echocardiography

A comprehensive resting echocardiography assessment was performed using commercially available equipment (GE E95, General Electric, Horten, Norway) (Mitchell et al., 2019). LV end‐diastolic and end‐systolic volumes and LV ejection fraction were measured according to the standard guidelines using the biplane Simpson's method of disks (Lang et al., 2015). LV‐focused views from an apical four chamber, two chamber and long axis view of at least three beats in duration were acquired at a frame rate of 60–90 frames/s for assessment of GLS. Furthermore, a 3D dataset incorporating all LV walls was acquired from an apical four chamber position with volume rate adjusted to at least 20 volumes/s. GLS and 3D volumes were calculated using dedicated analysis software on EchoPac (LV Automated Function Imaging analysis and Auto 4D LV analysis, V.204, General Electric, Horten, Norway). For GLS calculation, a region of interest was applied to the endocardial and epicardial borders of the LV with the tracking manually adjusted to cover the entire myocardium over the cardiac cycle. GLS was defined as the change in length in the longitudinal plane of the LV myocardium from end diastole to end systole over the 3 apical views. 3D volumes and LVEF were calculated from the 3D dataset with a region of interest applied to the endocardial border of the LV. This border was tracked by the software over the cardiac cycle and manual adjustment made where required. Participants were deemed inadequate for GLS or 3D assessment where two or more LV regions are unable to be visualized and tracked. One expert cardiac sonographer (RP) collected and analyzed all echocardiography data and has previously reported high intra‐observer reliability for GLS measurements (interclass correlation coefficient = 0.93) (Perry et al., 2020). The observer was blinded to group allocation during the collection and analysis of all echocardiography data.

#### 2.2.4 Exercise capacity

A symptom‐limited peak exercise test was performed on a Lode Corival (v5.4.0, Groningen, the Netherlands) electronically braked stationary recumbent cycle ergometer to measure VO_2_peak. The test followed an incremental ramp protocol, beginning at 25 watts (W) and increasing by 15 W each minute until volitional exhaustion. A 1‐min warm up and 3‐min cool‐down was completed at 0 W. Respiratory gas (O_2_ and CO_2_) was measured continuously via a facemask connected to a Parvomedics Trueone 2400 Metabolic Measurement System (Utah, United States). VO_2_peak was defined as the highest VO_2_ recorded before volitional exhaustion. Heart rate (HR) was measured using a Polar H10 (Kempele, Finland) wireless chest strap, and recorded in the last 10 s of each minute. Rating of perceived exertion (RPE) was measured using a Borg 6–20 RPE scale (Borg, 1998), and recorded in the last 10 s of each minute.

### 2.3 Exercise intervention

Participants in the exercise arm performed three aerobic exercise sessions per week, for eight consecutive weeks. Each session involved 30 min of continuous cycling on a Lode Corival (v5.4.0) electronically braked stationary recumbent cycle ergometer. Exercise intensity was prescribed using a percentage of participant's peak HR obtained during baseline VO_2_peak testing. The relative intensity of each session was moderate (between 60% and 70% HRpeak), with small progressions in intensity occurring every 2 weeks. All exercise sessions were delivered one‐to‐one and supervised by an accredited exercise physiologist. HR and RPE were recorded every 5 min to monitor exercise intensity and for safety purposes. Resting blood pressure or blood glucose was measured before and after each exercise session in participants with HTN or T2DM, respectively. All participants, regardless of their group (exercise or control), were instructed to continue with their daily activities as per normal and were not precluded from performing exercise independently of the study, with self‐reported physical activity recorded at each measurement timepoint using the International Physical Activity Questionnaire–Short Form (Craig et al., 2003) (see Section 2.2.1). Further, participants were encouraged to maintain their normal dietary habits throughout the duration of the study.

### 2.4 Sample size

The mean and standard deviation of GLS from each arm of the first three completed participants was used to retrospectively calculate the sample size, using a matched‐pair *t*‐test. With a standard deviation of the difference in means of 1.53 between groups, nine participants were required to provide 80% power to detect a mean difference of 1.7 between groups (10% relative change in GLS from a baseline GLS of −17%), with an alpha of 0.05. With inter‐participant variability reduced through the use of a cross over design (participants acting as their own control), smaller overall samples are expected (Alhadad et al., 2023; Savikj et al., 2019).

### 2.5 Statistical analysis

Continuous variables are expressed as mean ± standard deviation. A linear mixed effect model was used to compare outcomes between (exercise vs. control) and within (pre‐vs. post‐intervention) groups for echocardiographic and cardiorespiratory fitness variables. For all analyses, participant id was considered as a random effect. All participants who completed at least one arm of the trial were included in the analysis. Effect sizes (Cohen's *d*) and 95% confidence intervals (CI) for GLS, VO_2_peak, and systolic and diastolic blood pressure are presented and interpreted as 0.2 (small), 0.21–0.5 (medium), 0.51–0.8 (large) and 0.81–1.3 (very large) (Sullivan et al., 2012). Pearson's (*r*) correlation was performed to determine the association between relative change in GLS and VO_2_peak. All data were analyzed using Stata statistical software (v17.1, Stata Corp, TX, USA). Adherence to exercise training frequency was determined by calculating the ratio of attended exercise sessions to the total planned exercise sessions, expressed as a percentage. Adherence to prescribed exercise intensity was calculated by summing the average percentage of peak HR achieved across each individual exercise session divided by the total number of exercise sessions attended.

## 3 RESULTS

### 3.1 Participant characteristics

Fifteen women (aged 64.1 ± 4.7) with HTN (*n* = 12) and T2DM (*n* = 3) were recruited to participate, with nine completing both exercise and control arms. Four participants completed the control arm *only*, with two participants completing the exercise arm *only*. Reasons for not crossing over included illness unrelated to participation in the study (*n* = 4), an inability to commit to the exercise intervention (*n* = 1) and unforeseen family circumstances (*n* = 1). Baseline characteristics for each condition are outlined in Table 1. The average washout period for participants that completed the crossover was 21.6 ± 14.2 (range 4–46) weeks. There was a high degree of variability in the washout period due to participant availability.

**TABLE 1:** Baseline characteristics of participants.

	Exercise (*n* = 11)	Control (*n* = 13)
Age (years)	62.6 ± 4.7	65.4 ± 4.6
Height (cm)	158.7 ± 5.8	159.6 ± 7.7
Weight (kg)	78.9 ± 16.6	77.8 ± 12.6
Body mass index (kg/m^2^)	31.1 ± 5.4	30.4 ± 3.9
Cardiovascular risk factors		
Hypertension, *n* (%)	8 (72.7)	10 (76.9)
Type 2 diabetes, *n* (%)	3 (27.3)	3 (23.1)
Dyslipidaemia, *n* (%)	4 (36.4)	5 (38.5)
Overweight–BMI >25, *n* (%)	9 (81.8)	13 (100)
Obese–BMI >30, *n* (%)	7 (63.6)	8 (61.5)
Medications		
Angiotensin receptor blocker (*n*)	4	5
ACE inhibitor (*n*)	3	3
Calcium channel blockers (*n*)	1	‐
Diuretic (*n*)	1	1
Metformin (*n*)	3	3
Gliclazide (*n*)	2	2
Smoking history		
Current (*n*)	0	0
Previous (*n*)	2	3
Never (*n*)	9	10
Self‐reported physical activity		
Vigorous (mins/week)	44.1 ± 68.4	25.4 ± 69.1
Moderate (mins/week)	74.6 ± 91.2	128.8 ± 125.4
Walking (mins/week)	210 ± 126.2	198.7 ± 110.2
Sedentary time (mins/day)	267.3 ± 126.2	327.7 ± 152.2

Abbreviations: %, percent per group; ACE, angiotensin converting enzyme; BMI, body mass index; cm, centimeters; kg, kilograms; kg/m^2^, kilograms per meter squared; mins/day, minutes per day; mins/week, minutes per week; *n*, number of participants.

### 3.2 Exercise intervention adherence

Participants attended 21 ± 3.8 (range 14–24) exercise sessions over the 8‐week intervention period (86.6% adherence to prescribed training frequency). Adherence to prescribed exercise intensity (60%–70% HRpeak) was good, with an average exercise intensity completed by participants of 69 ± 0.03% (range 65%–73%) of HRpeak. No serious adverse events (defined as events resulting in death, persistent or significant disability/incapacity, life‐threatening situations or requiring inpatient hospitalization or prolongation of existing hospitalization) occurred during exercise in any participants. One participant experienced an asymptomatic reduction in blood glucose below 5.5 mmol/L, following a single exercise session.

### 3.3 CV function and cardiorespiratory fitness outcomes

Table 2 presents the echocardiography and cardiorespiratory fitness outcomes for exercise and control arms. One participant was excluded from analysis of echocardiography parameters (including GLS) due to poor image quality.

**TABLE 2:** Resting echocardiography and cardiorespiratory fitness outcomes.

	Exercise (*n* = 11)			Control (*n* = 13)			*p* (between groups)
	Baseline	8‐week	*p* (within group)	Baseline	8‐week	*p* (within group)	
GLS (%)	−16.8 ± 1.48	−18.4 ± 1.78	**<0.001**	−17.16 ± 1.99	−16.91 ± 1.44	0.585	**<0.001**
LV ejection fraction (%)	59.5 ± 4.55	58.8 ± 3.46	0.683	61.0 ± 5.54	59.7 ± 4.60	0.498	0.810
Stroke volume (mL)	72.2 ± 21.06	75.8 ± 20.66	0.475	71.4 ± 15.22	66.4 ± 15.19	0.322	0.230
Cardiac output (L/min)	4.72 ± 1.34	4.94 ± 1.38	0.590	4.78 ± 0.90	4.37 ± 1.17	0.226	0.232
Weight (kg)	78.9 ± 16.6	78.5 ± 15.9	0.263	77.8 ± 12.6	77.7 ± 12.92	0.821	0.569
VO_2_peak (mL/kg/min)	17.99 ± 2.13	19.23 ± 2.58	**<0.001**	17.55 ± 2.71	17.22 ± 2.71	0.269	**0.011**
VO_2_peak (L/min)	1.40 ± 0.25	1.49 ± 0.24	**<0.001**	1.35 ± 0.22	1.33 ± 0.23	0.535	**0.012**
Systolic BP (mmHg)	137.4 ± 15.0	122.8 ± 13.6	**<0.001**	133.6 ± 15.0	129.4 ± 17.7	0.340	0.078
Diastolic BP (mmHg)	87.6 ± 10.3	79.6 ± 6.5	**<0.001**	83.9 ± 8.0	84.6 ± 11.4	0.770	**0.012**

*Note*: *n = 10 and *n* = 12 for exercise and control arms respectively for echocardiography outcomes measures.

Abbreviations: BP, blood pressure; GLS, global longitudinal strain; kg, kilograms; l/min, liters per minute; LV, left ventricular; mL, milliliters; mL/kg/min, milliliters per kilogram per minute; mmHg, millimeters of mercury; *n*, number of participants; VO_2_peak, peak oxygen consumption.

#### 3.3.1 Primary outcomes

GLS (%) improved significantly following 8 weeks of exercise (mean change 1.6 ± 0.92, 95% CI = 1.03, 2.16; *d* = 0.98 [*very large effect*], *p* ≤ 0.001), with no significant changes observed following the control period (mean change −0.25 ± 1.59, 95% CI = −1.15, 0.65; *d* = −0.14 [*no effect*], *p* = 0.585). This change was significantly different between groups (mean change 1.85 ± 2.04, 95% CI = 0.78, 2.92; *d* = 1.02 [*very large effect*], *p* ≤ 0.001).

#### 3.3.2 Secondary outcomes

VO_2_peak (mL/kg/min) increased significantly following 8 weeks of exercise (mean change 1.24 ± 1.13, 95% CI = 0.57, 1.90; *d* = 0.53 [*moderate effect*], *p* ≤ 0.001), with no change observed following the control period (mean change −0.33 ± 1.08, 95% CI = −0.92, 0.26; *d* = −0.12 [*no effect*], *p* = 0.269). This change was significantly different between groups (mean change 1.56 ± 2.40, 95% CI = 0.36, 2.78; *d* = 0.63 [*moderate effect*], *p* = 0.011).

Systolic and diastolic blood pressure decreased significantly following 8 weeks of exercise (mean change −14.6 ± 12.64, 95% CI = −22.0, −7.01; *d* = −1.02 [*very large effect*], *p* ≤ 0.001—mean change −8.0 ± 6.99, 95% CI = −12.0, −3.78; *d* = −0.93 [*very large effect*], *p* ≤ 0.001, respectively), with no change observed following the control period (mean change −4.2 ± 15.98, 95% CI = −12.92, −4.45; *d* = −0.26 [*small effect*], *p* ≤ 0.340—mean change 0.7 ± 8.56, 95% CI = −3.96, 5.34; *d* = 0.07 [*no effect*], *p* ≤ 0.770, respectively). This change was significantly different between groups for diastolic blood pressure (mean change −8.6 ± 13.27, 95% CI −15.31, −1.89; *d* = −0.92 [*very large effect*], *p* = 0.012), but not systolic blood pressure (mean change −10.4 ± 22.68, 95% CI = −21.79, 1.16; *d* = −0.67 [*moderate effect*], *p* = 0.078).

No changes in LV ejection fraction, stroke volume, cardiac output or bodyweight were observed within or between groups (Table 2).

**FIGURE 1:**
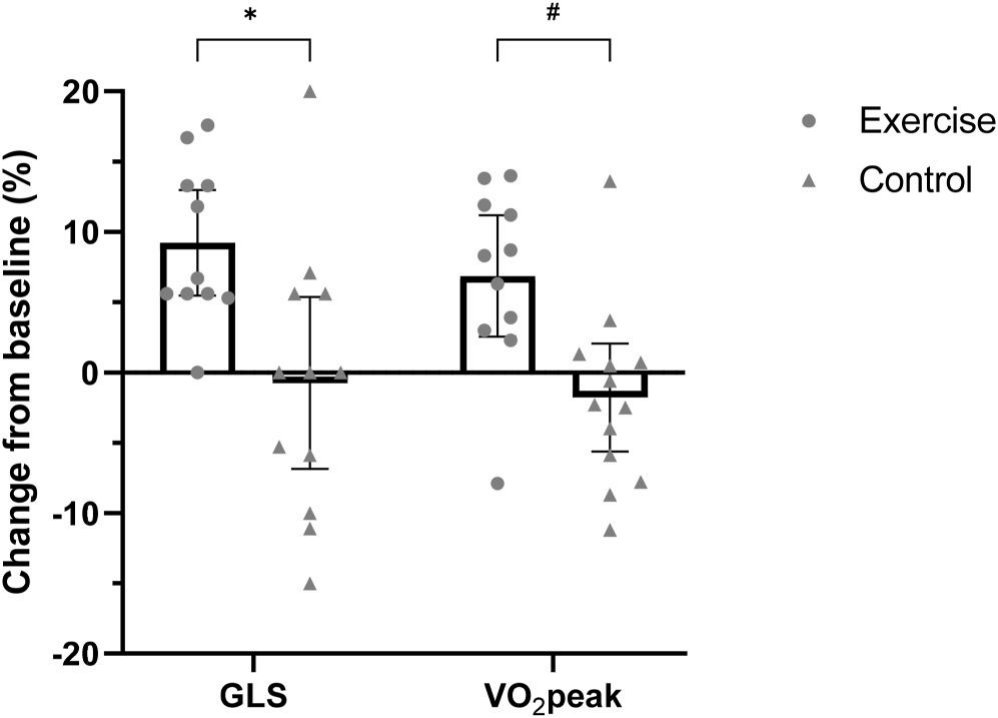
Mean relative change (with 95% confidence intervals) in GLS and VO_2_peak from baseline to 8 weeks in exercise and control arms. Individual data points represent relative change in GLS and VO_2_peak from baseline to 8 weeks for each individual participant. A relative increase in GLS of 9.56% was observed following exercise (*p* ≤ 0.001), with no change observed following the control period (*p* = 0.585). Similarly, VO_2_peak increased by 6.86% from baseline following exercise (*p* ≤ 0.001), with no change following the control period (*p* = 0.269). *Group × time effect for GLS, *p* ≤ 0.001. ^#^Group × time effect for VO_2_ peak, *p* = 0.011.

Figure 1 illustrates the mean *relative* change in GLS and VO_2_peak from baseline to 8 weeks in exercise and control arms, with each individual data point representing the relative change for each individual participant. A relative increase in GLS of 9.56% was observed following exercise, with a relative decrease of 1.46% observed following the control period. Similarly, VO_2_peak increased by 6.86% from baseline following exercise, with a 1.88% decrease observed following the control period. GLS did not decrease from baseline in any participants following exercise. Improvements in VO_2_peak from baseline were observed in 10 of the 11 participants that completed the exercise intervention, with reductions in VO_2_peak from baseline observed in eight of 13 participants following the control period.

**FIGURE 2:**
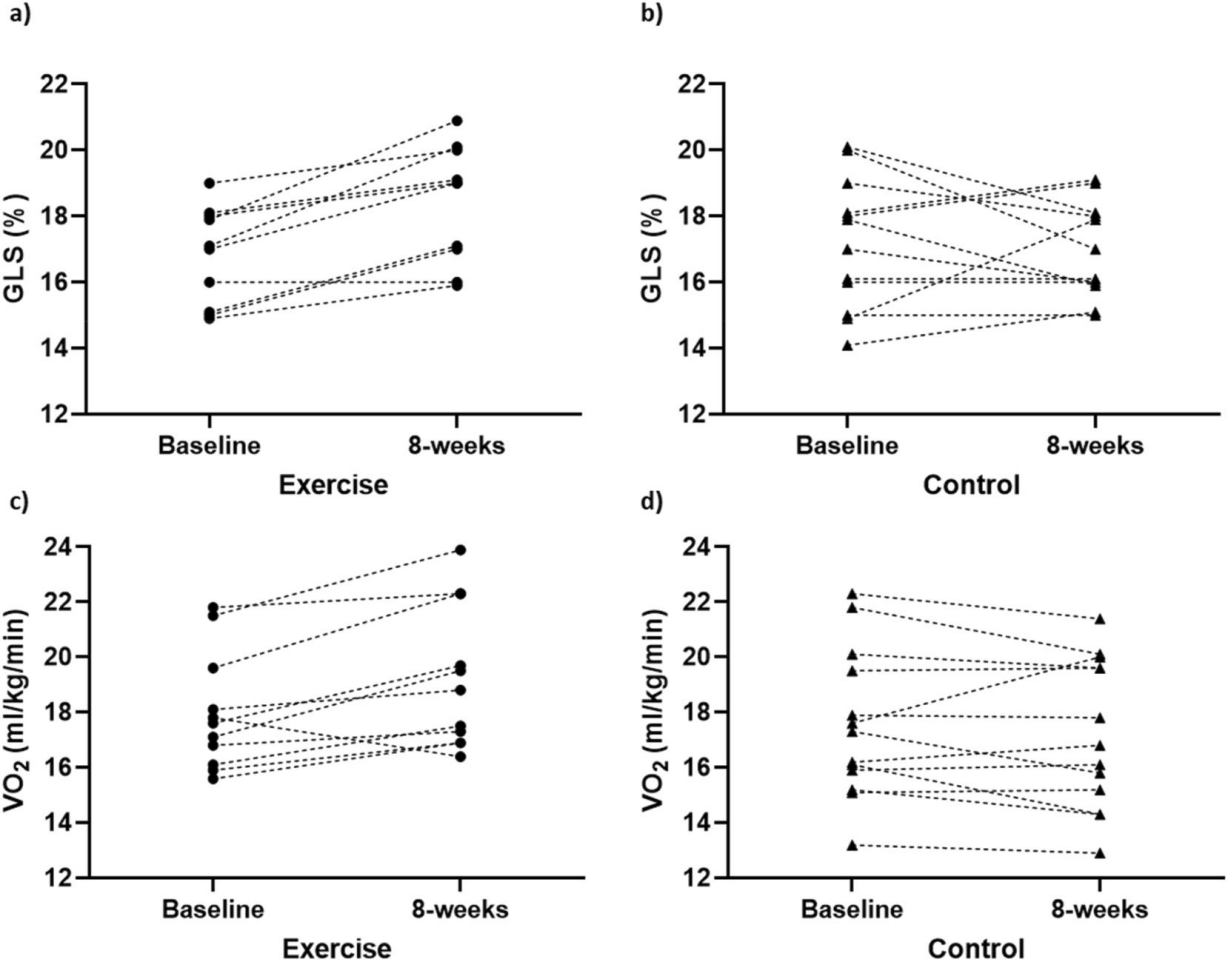
Absolute change in GLS (A, B) and VO_2_peak (C, D) from baseline to 8 weeks for each individual participant in exercise (A, C) and control (B, D) groups. Each connected data point represents an individual participant.

Figure 2A–D depicts the *absolute* change in GLS and VO_2_peak from baseline to 8 weeks for each individual participant following exercise and control periods.

### 3.4 Correlation analyses

There was no significant association observed between relative change in GLS and relative change in VO_2_peak (*r* = 0.386; *p* = 0.084).

## 4 DISCUSSION

This study explored the impact of exercise on GLS and VO_2_peak in women with HTN and T2DM. Both GLS and VO_2_peak improved significantly following exercise, with no change observed after control. Despite observing a significant improvement in both GLS and VO_2_peak following exercise, there was no significant association between changes in the two variables. Findings from this research provide insight into the ability of GLS to measure the effectiveness of aerobic exercise interventions on myocardial function in women at risk of developing CV disease.

As GLS is a sensitive measure of myocardial function, and aerobic exercise is recognised to improve CV health and function (Kr et al., 2019), it was hypothesized that exercise would positively impact GLS in women with HTN and T2DM. Results support that this hypothesis, with an average of 9.56% relative increase in GLS observed following 8 weeks of exercise, compared to a 1.46% relative decrease following the control period. With reductions in GLS a strong prognostic indicator for future CV abnormalities (D’Elia et al., 2020; Fortuni et al., 2021; Kalam et al., 2014), the significant improvement in GLS supports the use of aerobic exercise to improve myocardial function and reduce CV disease risk. In a recently published work, O’Driscoll et al. (O’Driscoll et al., 2022) reported a 13.3% relative increase in GLS, observed following 4 weeks of isometric exercise training in hypertensive males, was associated with a >24% reduced risk of all‐cause mortality, and >32% lower risk of heart failure. As such, the 9.56% relative increase in GLS in this current study may infer a clinically significant benefit of aerobic exercise for women with HTN and T2DM. Despite findings from O’Driscoll et al. (O’Driscoll et al., 2022), the exact clinical benefit of increases in GLS has not been formally defined in the literature. In addition to GLS, a 1 mL/kg/min increase in VO_2_peak is associated with an 11% and 15% reduction in all‐cause and CV disease mortality, respectively (Imboden et al., 2019). As such, the 1.24 mL/kg/min increase in VO_2_peak observed following exercise, alongside a 9.56% relative increase in GLS, suggests a substantial clinical benefit of exercise on CV health and function for women with HTN and T2DM. Significant reductions in systolic and diastolic blood pressure (*p* < 0.001 for both) were also observed following exercise (Table 2), which may indicate another clinically relevant finding given that GLS has been shown to be impaired in individuals with HTN and T2DM (Soufi Taleb Bendiab et al., 2017; Tadic et al., 2021). A GLS of −18% is considered the lower limit of normal in women (Asch et al., 2019). Mean GLS improved from −16.8% to −18.4% following exercise, with six of the 10 participants having a GLS value of −18% or greater, and two improving GLS from below −18% to above −18% upon the completion of exercise (figure 2A).

Despite observing positive changes in both GLS and VO_2_peak, there was no significant association between changes in the two variables. Factors contributing to changes in VO_2_peak can be both central (i.e., change in cardiac output) and peripheral (i.e., change in the ability of muscles to extract oxygen) (Lee et al., 2021). In comparison, GLS is specific to longitudinal changes in the myocardium. Therefore, while GLS and VO_2_peak are both strong indicators of mortality, and linked closely to CV disease and dysfunction, the mechanisms associated with their change are different, which may explain the lack of correlation observed.

Results demonstrate the ability of GLS to measure improvements in myocardial function following aerobic exercise in women at risk of developing CV disease. Given its sensitivity, exercise professionals working in multi‐disciplinary settings (i.e., hospitals) could use GLS, alongside other traditional exercise measures (i.e., VO_2_peak), to gain feedback on the effectiveness of an exercise intervention in its early stages. Symptom limited exercise testing, to determine VO_2_peak, is also not readily available in most clinical settings due to associated costs and technical expertise required, adding further potential benefit to the use of GLS data in exercise settings where advanced echocardiography is available.

Importantly, no participant experienced a reduction in GLS from baseline following exercise (Figures 1 and 2A), with no adverse events occurring during any exercise session. This further highlights the safety of exercise for women with HTN or T2DM, and aligns with previous research suggesting that exercise does not negatively impact GLS (Murray et al., 2022). One participant experienced a reduction in VO_2_peak following exercise, in comparison to eight participants following the control period. The reduction observed following exercise is likely a result of this participant attending 14 of the 24 available exercise sessions. The large variability in individual changes in GLS and VO_2_peak observed following the control period (Figures 1 and 2B,D) may be explained by not precluding participants from performing exercise or physical activity outside of the study. However, the constant, positive response observed in GLS and VO_2_peak following 8 weeks of exercise suggests that the impact of exercise on GLS in this cohort was real.

Despite a significant improvement in GLS following exercise, no change in LV ejection fraction was observed in either group (Table 2). LV ejection fraction is a common measure used to diagnose CV dysfunction in a range of populations (McDonagh et al., 2021; Zamorano et al., 2017); however, it has been shown to be insensitive to changes of <10% points (Marwick, 2013). The lack of change in LV ejection fraction observed between groups in this study is likely explained by this insensitivity, and suggests that GLS may be superior for detecting improvements in myocardial function following short‐term exercise interventions. This seems reasonable when considering there is evidence supporting the greater prognostic ability of GLS in comparison to LV ejection fraction for predicting adverse cardiac events (Kalam et al., 2014). Findings from this study may also have implications for other high CV disease risk populations, including individuals undergoing cardiotoxic chemotherapy for the treatment of breast cancer, supporting the need for further research investigating the effect of exercise on GLS in this population (Thavendiranathan et al., 2014).

### 4.1 Strengths, limitations and future research

The presentation of individual data, clear explanation of exercise interventions and echocardiography methodologies and reporting of exercise adherence are all strengths of this study—something that the literature in this field to date has been criticized for (Murray et al., 2022). The findings of this research also add important information to the literature in an underrepresented cohort in this field, that being females with CV disease risk factors. Being a crossover design, the authors acknowledge the potential carryover of physiological adaptations in those who were randomized to the exercise arm first as a limitation of the study. However, with both a 3‐ and 4‐week washout period previously shown to be adequate for measures of CV function and fitness to return to baseline following an exercise intervention (O’Driscoll et al., 2018; O’Driscoll et al., 2022), and the washout period in this study being 21.6 ± 14.2 (range 4–46) weeks, this is unlikely to have impacted the outcomes of this research. A true measure of VO_2_ maximum would have provided greater insight into the effect of exercise on cardiorespiratory fitness. However, this testing was not deemed appropriate in this population due to the requirement of a validation stage, which adds additional participant burden. As the clinical benefit of improvements in GLS has not been defined in the literature, future research may investigate the extent to which improvements in GLS reduce the risk of CV disease and dysfunction. Given its sensitivity, future research may also consider monitoring GLS more frequently throughout exercise interventions (i.e., every 2‐week) to inform exercise professionals regarding the effect of exercise interventions on myocardial function in its acute stages. This will allow for modifications to exercise programing if required, maximizing participant outcomes.

## 5 CONCLUSION

Aerobic exercise positively impacts GLS and VO_2_peak in women with HTN and T2DM. Given its sensitivity, GLS may inform exercise professionals regarding the effectiveness of an aerobic exercise intervention in its early stages, alongside changes in traditional exercise measures (VO_2_peak). Future research should investigate the extent to which improvements in GLS reduce the risk of CV disease and dysfunction.

## AUTHOR CONTRIBUTIONS

James Murray, Hunter Bennett, Eva Bezak, and Rebecca Perry contributed to the conception and design of the work. All authors contributed to the analysis and interpretation of data. James Murray, Hunter Bennett, and Rebecca Perry drafted the manuscript. Hunter Bennett, Eva Bezak, and Rebecca Perry critically reviewed the manuscript. All authors gave approval and agreed to be accountable for all aspects of the work ensuring integrity and accuracy.

## CONFLICT OF INTEREST STATEMENT

The authors declare that there is no conflict of interest.

## Supporting information

Supporting Information S1
