# Supplementary material for: Exercise positively impacts global longitudinal strain in women at risk of developing cardiovascular disease
Source: Eur J Sport Sci. 2024 Mar 18;24(5):527–36. doi: 10.1002/ejsc.12047 (PMC11235825; doi:10.1002/ejsc.12047)
Supplement: Supplementary file 1 — Supporting Information S1 [file EJSC-24-527-s001.docx]

**Supplementary Material**

**List of Contents (in order of appearance):**

**Table 1.** Additional resting echocardiography parameters.

**Table 2.** Additional cardiorespiratory fitness and symptom limited exercise test parameters.

**Figure 1.** Overview of randomised crossover design and timepoints of outcome measure acquisition.

**Table 1.** Additional resting echocardiography parameters**.**

|  | Exercise | | | |  | Control | | | |  |  |
| --- | --- | --- | --- | --- | --- | --- | --- | --- | --- | --- | --- |
|  | n | Baseline | 8-weeks | *p* (within group) |  | n | Baseline | 8-weeks | *p* (within group) |  | *p* (between groups) |
| 2D EDV (mL) | 10 | 87.9 ± 20.0 | 78.3 ± 19.8 | **0.015** |  | 12 | 75.8 ± 16.1 | 76.4 ± 13.7 | 0.871 |  | 0.146 |
| 2D ESV (mL) | 10 | 35.8 ± 8.1 | 34.7 ± 13.5 | 0.683 |  | 12 | 29.8 ± 6.8 | 30.3 ± 5.9 | 0.821 |  | 0.638 |
| LA Strain Res (%) | 10 | 23.9 ± 3.6 | 25.6 ± 4.4 | 0.313 |  | 12 | 24.3 ± 7.8 | 23.8 ± 5.3 | 0.777 |  | 0.411 |
| LA Strain Cond (%) | 10 | -10.2 ± 3.2 | -11.1 ± 2.7 | 0.430 |  | 12 | -10.4 ± 4.5 | -10.3 ± 4.1 | 0.945 |  | 0.571 |
| LA Strain Ct (%) | 10 | -13.4 ± 4.3 | -14.9 ± 4.8 | 0.434 |  | 12 | -14 ± 5.3 | -13.3 ± 5.1 | 0.656 |  | 0.349 |
| RV FWS (%) | 7 | -19.7 ± 3.7 | -21.5 ± 2.5 | 0.220 |  | 8 | -20.3 ± 4.3 | -18.4 ± 4.5 | 0.308 |  | 0.139 |

n – number of participants. 2D – two dimensional. EDV – end diastolic volume. ESV – end systolic volume. mL – millilitres. LA – left atrial.
Res – reservoir. Cond – conduit. Ct – contractile. RV FWS – right ventricular free wall strain.
* n reflects the number of participants included in analysis of each echocardiography parameter, as certain participants were excluded from
analysis due to poor image quality.

**Table 2.** Additional cardiorespiratory fitness and symptom limited exercise test parameters.

|  | Exercise (n=11) | | |  | Control (n=13) | | |  |  |
| --- | --- | --- | --- | --- | --- | --- | --- | --- | --- |
|  | Baseline | 8-weeks | *p* (within group) |  | Baseline | 8-weeks | *p* (within group) |  | *p* (between groups) |
| Resting HR (bpm) | 71.6 ± 7.3 | 75.1 ± 11.7 | 0.314 |  | 74.5 ± 8.6 | 73 ± 11.2 | 0.373 |  | 0.178 |
| Peak HR (bpm) | 140.2 ± 14.1 | 143.5 ± 10.5 | 0.106 |  | 139.1 ± 12.6 | 139.3 ± 10.5 | 0.946 |  | 0.258 |
| Peak power (watts) | 101.6 ± 18.8 | 113.6 ± 17.0 | **<0.001** |  | 100 ± 23.7 | 101.2 ± 21.6 | 0.738 |  | 0.078 |
| Peak RPE | 18.3 ± 1.4 | 15.6 ± 2.2 | **<0.001** |  | 16.5 ± 2.2 | 16.5 ± 1.8 | 1.000 |  | **0.001** |
| RER | 1.16 ± 0.1 | 1.15 ± 0.1 | 0.221 |  | 1.14 ± 0.1 | 1.13 ± 0.1 | 0.445 |  | 0.959 |

n = number of participants. HR – heart rate. bpm – beats per minute. RPE – ratings of perceived exertion. RER – respiratory exchange ratio.


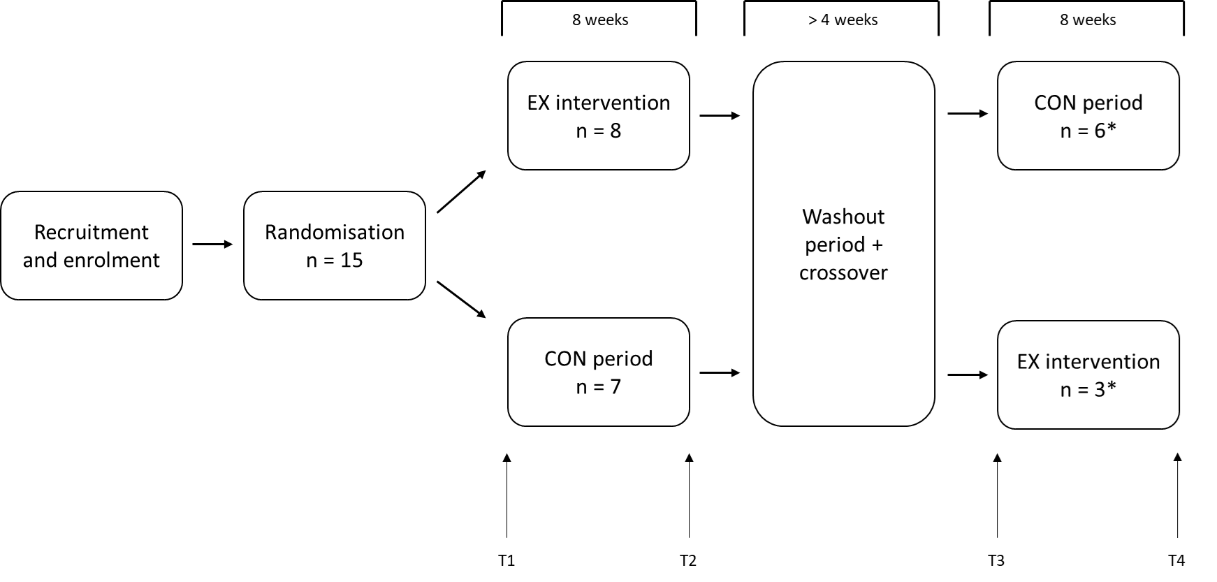


**Figure 1.** Overview of randomised crossover design and timepoints of outcome measure acquisition.
T - timepoint of outcome measure acquisition. n - number of participants in respective group. EX – exercise. CON – control. *Not all participants returned following washout period for reasons outlined in section 3.1. of the manuscript.
